# Transcription factor 21 expression in injured podocytes of glomerular diseases

**DOI:** 10.1038/s41598-020-68422-3

**Published:** 2020-07-13

**Authors:** Joichi Usui, Misa Yaguchi, Satoshi Yamazaki, Mayumi Takahashi-Kobayashi, Tetsuya Kawamura, Shuzo Kaneko, Surya V. Seshan, Pierre Ronco, Kunihiro Yamagata

**Affiliations:** 1 Department of Nephrology, Faculty of Medicine, University of Tsukuba, 1-1-1 Tennodai, Tsukuba, Ibaraki 305-8575 Japan; 2 Laboratory of Stem Cell Therapy, Faculty of Medicine, University of Tsukuba, Tsukuba, Ibaraki 305-8576 Japan; 3 Department of Pathology and Laboratory Medicine, Weill Cornell Medicine, New York, NY 10065 USA; 4 Division of Stem Cell Therapy, Distinguished Professor Units, The Institute of Medical Science, The University Tokyo, Tokyo, 108-8639 Japan; 5 Université Pierre Et Marie Curie Paris 06, Institut National de la Santé et de la Recherche Médicale, Unité Mixte de Recherche S1155, SorbonneUniversité, Paris, France

**Keywords:** Nephrology, Kidney diseases

## Abstract

Transcription factor 21 (TCF21) is one of the essential transcription factors in kidney development. To elucidate its influence on glomerular disease, we have investigated TCF21 expression in human and rat kidney tissue, and its urinary concentration. Immunohistological analysis suggested the highest TCF21 expression in nephrotic syndrome along with the urinary protein level. Urinary TCF21 concentration in human showed a positive correlation with its podocyte expression level. In nephrotic rat models, TCF21 expression in podocytes increased along with the severity of nephrotic syndrome. Next, in vitro experiments using Tcf21-expressing murine podocyte cell line, we could observe some Tcf21-dependent effects, related with actin cytoskeleton dysregulation and apoptosis. Our study illustrated TCF21 expression changes in vivo and its in vitro-functional significance injured podocytes.

## Introduction

Kidney development requires the contribution and coordination of multiple master genes. Among them, several transcription factors, which are responsible for both podocyte differentiation and morphogenesis, contribute to the development of proteinuria due to podocyte dysregulation. As we mention below, Wilms tumor suppressor gene (*Wt1*) and V-maf musculoaponeurotic fibrosarcoma oncogene homolog B (*Mafb*) are examples of such factors that regulate podocyte development^1,2^. As a first example, *Wt1* expressed in the metanephric mesenchyme of mammals is identified as a key transcription factor in developing metanephros^3^. In humans, *WT1* abnormality has been reported to cause Denys-Drash syndrome (DDS), which presents with genetic steroid resistant nephrotic syndrome^1^. In addition, adult *Wt1* conditional knockout mice developed a glomerular injury resembling human DDS^4^. A second example, the *Mafb* gene, also known as *Kreisler/Krml1*, is expressed in podocytes in the latter stage of metanephric development　and participates in the formation of the foot process^2,5^. Adult *Mafb* knockout mice exhibit diffuse foot processes effacements with nephrin deficiency and may ultimately develop congenital nephrotic syndrome. Moreover, adult *Mafb* overexpression mice protect podocytes in diabetic kidney disease model^6^. Recently in humans, *MAFB* mutation was reported to develop Duane Retraction syndrome with focal segmental glomerulosclerosis (FSGS)^7^.

In addition to *Wt1* and *Mafb*, here we present transcription factor 21 (*Tcf21*, also called *Pod1/Capsulin/Epicardin*), belonging to the basic-helix loop helix (bHLH) family, as a key factor in kidney development^8^. *Tcf21* is expressed in the mesenchyme of multiple organs such as the kidney, heart and lung and plays an essential role in organ morphogenesis^9^. During metanephrogenesis, *Tcf21* first appears on E10.5 mouse mesenchymal cells, and is then expressed in nephron progenitor cells in the later stage, such as s-shaped body stage^8,10^. During late nephrogenesis, its expression is limited to interstitial cells and podocytes differentiated from nephron progenitor cells. A lack of *Tcf21* results in hypoplastic metanephros, which is related to poor differentiation from the metanephric mesenchyme to nephron progenitor cells, and also to growth failure in ureteric buds. In the later stage in *Tcf21* knockout mice, the foot processes, required for the regulation of proteinuria, becomes aberrant in glomerular podocytes. As adult disease model, *Tcf21* knockout adult mice showed the development of FSGS with age, and the DKD models changed for the worse^11^. Even though these results have been established in mice, scant data exist for humans. The role of TCF21 in humans throughout the whole course from the embryonic stage to the adult stage remains to be established.

We hypothesized that TCF21 is involved in the development of proteinuria as are other key transcription factors. Herein we clarify the contributions of TCF21 to the regulation of proteinuria by detecting podocyte-expressed TCF21 in various glomerular diseases and evaluating its in- vitro effects.

## Results

### Histological analysis of TCF21 in human glomerular diseases and rat nephrosis model

In preparation for this immunohistochemistry, we verified the specificity of primary antibody by using the antibody-absorption method reacted with recombinant human TCF21 peptide antigen. The stain of the supernatant reacted with antigen peptide was not found in NGD, IgAN and MGN samples, comparing to the positive stain of the anti-TCF21 antibody usage (Supplementary Fig. 1a–c). Therefore, the primary antibody specifically recognized in human TCF21 protein.

**Figure 1:**
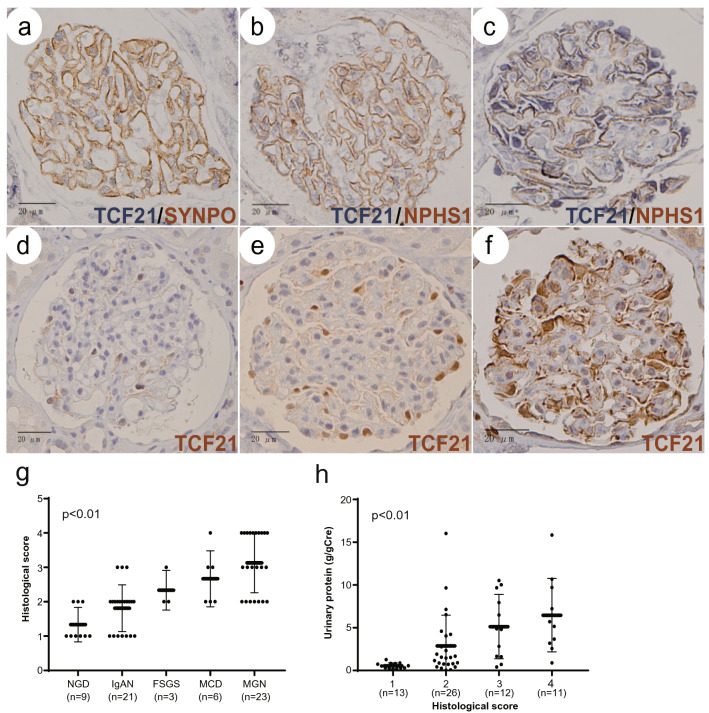
Histological analysis of TCF21 in human glomerular diseases. **a**, **b** The human normal glomeruli showed that TCF21 weakly expressed in the nuclei of podocytes linear marking along with synaptopodin (SYNPO, **a**) and nephrin (NPHS1, **b**). **c** In patients with nephrotic syndrome, MGN, NPHS1-stain somewhat decreased, but TCF21 highly expressed in the cellular body of podocytes along the lateral side of NPHS1-positivity. **d** In the sample of NGD defined score 1, TCF21 weakly expressed in the nuclei of segmental podocytes. **e** In IgAN sample defined score 2, TCF21 expressed in the nuclei of global podocytes. **f** In nephrotic syndrome, MGN sample defined score 4, TCF21 expression globally expanded in the cytoplasm of podocytes. **g** The comparison of TCF21 histological score among various glomerular diseases. Their groups were statistically significant among each glomerular disease (p < 0.01). **h** The relationship between TCF21 histological score and urinary protein level. The urinary protein level was also statistically significant among each histological TCF21 expression level (p < 0.01). Statistical analysis was performed with the Kruskal–Wallis test (**g**, **h**).

First, we histologically investigated the expression and localization of TCF21 in human normal glomeruli and various glomerular diseases using double immunohistochemistry. The human normal glomeruli showed that TCF21 was weakly expressed in the nuclei of podocytes localized by specific antigens, such as synaptopodin (SYNPO) and nephrin (NPHS1) (Fig. 1a, b). The nephrotic sample appeared that TCF21 extended to express in the cytoplasm of podocytes (Fig. 1c). Therefore, we found that TCF21 expressed in podocytes of both normal and injured glomeruli. Additionally, we observed the low-power magnification view of the sections. TCF21 mainly expressed with podocytes of glomeruli, rather than tubulointerstitial cells in most sections (Supplementary Fig. 2a, b). To support this result, we used web-based mRNA expression database, Nephroseq datasets, and compared *TCF21* mRNA expression between glomeruli and tubulointerstitium. The *TCF21* expression level in glomeruli was much higher than that in tubulointerstitium (Supplementary Fig. 3a). Next, we semi-quantitatively analyzed TCF21 expression using single immunohistochemistry and compared expression level among various glomerular diseases. As mentioned above, the normal glomeruli showed that TCF21 weakly expressed in the nuclei of podocytes (Fig. 1d). The glomeruli of patients with mild proteinuria, such as IgAN, revealed that the TCF21 expression in the nuclei of podocytes increased (Fig. 1e). Additionally, the glomeruli of patients with nephrotic syndrome revealed that the TCF21 expression in podocytes increased involving the nucleus, the cytoplasm and dendrite (Fig. 1f). Then, the TCF21 expression in podocytes was semiquantitatively evaluated and compared to the magnitude of urinary protein or glomerular diseases. To compare histological TCF21 expression in podocytes among NGD and glomerular diseases, their groups were statistically significant among each glomerular disease (Fig. 1g, p < 0.01, based on urinary protein; 0.4 ± 0.4 g/gCre in NGD, 1.1 ± 0.7 g/gCre in IgAN, 5.5 ± 1.4 g/gCre in FSGS, 5.7 ± 1.9 g/gCre in MCD and 6.0 ± 4.7 g/gCre in MGN). Conversely, the comparison of the level of urinary protein to histological TCF21 expression levels was also statistically significant (Fig. 1h, p < 0.01). To support this result, we also used Nephroseq datasets, and compared *TCF21* mRNA expression among some glomerular diseases including healthy living donor, MGN and MCD. Although this datasets did not provide statistical analysis, *TCF21* mRNA expression in MCD and MGN samples were somewhat higher than that in healthy living donor (Supplementary Fig. 3b). Additionally, we observed the TCF21 expression of podocytes in rat nephrotic models. The TCF21 expression of nuclei of podocytes in the nephrotic phase (score 2 in all examined glomeruli) was higher than that of the control (score 0 in all examined glomeruli) and then the TCF21 expression returned to weak level (score 0 in all examined glomeruli) in the recovery phase (Supplementary Fig. 4a-d). Therefore, we confirmed that TCF21 is highly expressed in podocytes of both human and rat glomeruli with nephrotic syndrome.

**Figure 2:**
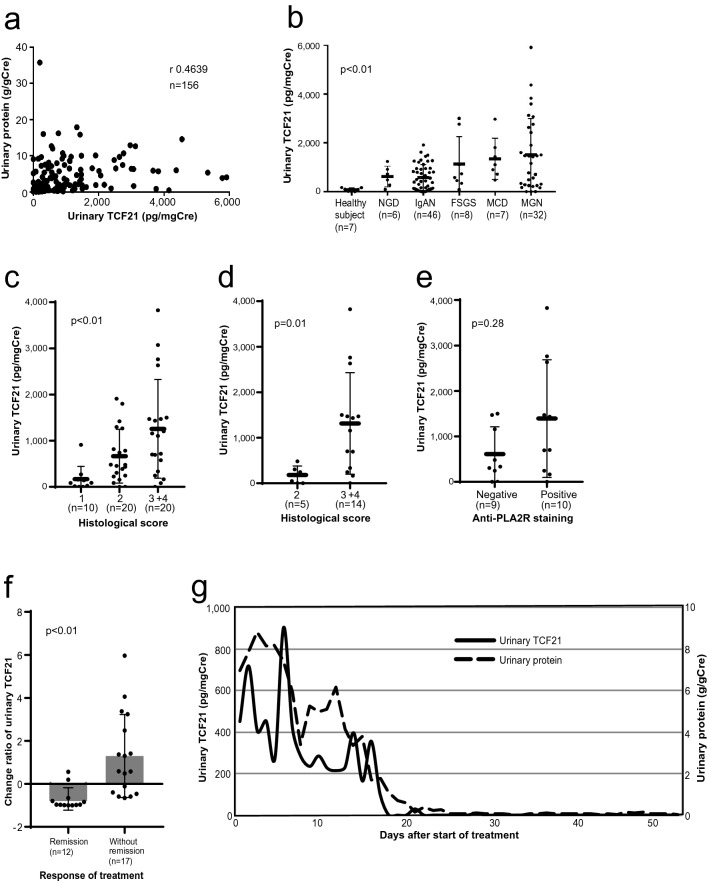
Urinary TCF21 levels in human glomerular diseases. **a** Urinary TCF21 levels showed a weak positive correlation with urinary protein levels (r 0.4639). **b** Urinary TCF21 concentration was statistically significant among each glomerular disease, and higher in nephrotic syndrome (p < 0.01). **c**, **d** Urinary TCF21 levels were positively associated with TCF21 histological score in total 50 cases (**c**) and 19 cases with MGN (**d**) (c: p < 0.01, d: p = 0.01, respectively). **e** The comparison of urinary TCF21 levels in MGN patients with or without anti-PLA2R positivity. It was not statistically significant (p = 0.28). **f** The change ratio of urinary TCF21 in treatment response of nephrotic patients. The change ratio was calculated from urinary TCF21 level at pre-/post-treatment. It was downward greater in remission group than in non-remission group (p < 0.01). **g** Continuous urinary TCF21 and protein levels during the remission-induction treatment in a case of steroid-sensitive MCD. Statistical analysis was performed with Spearman’s rank correlation coefficient (**a**), the Kruskal–Wallis test (**b**, **c**) and Mann Whitney *U* test (**d**–**f**).

**Figure 3:**
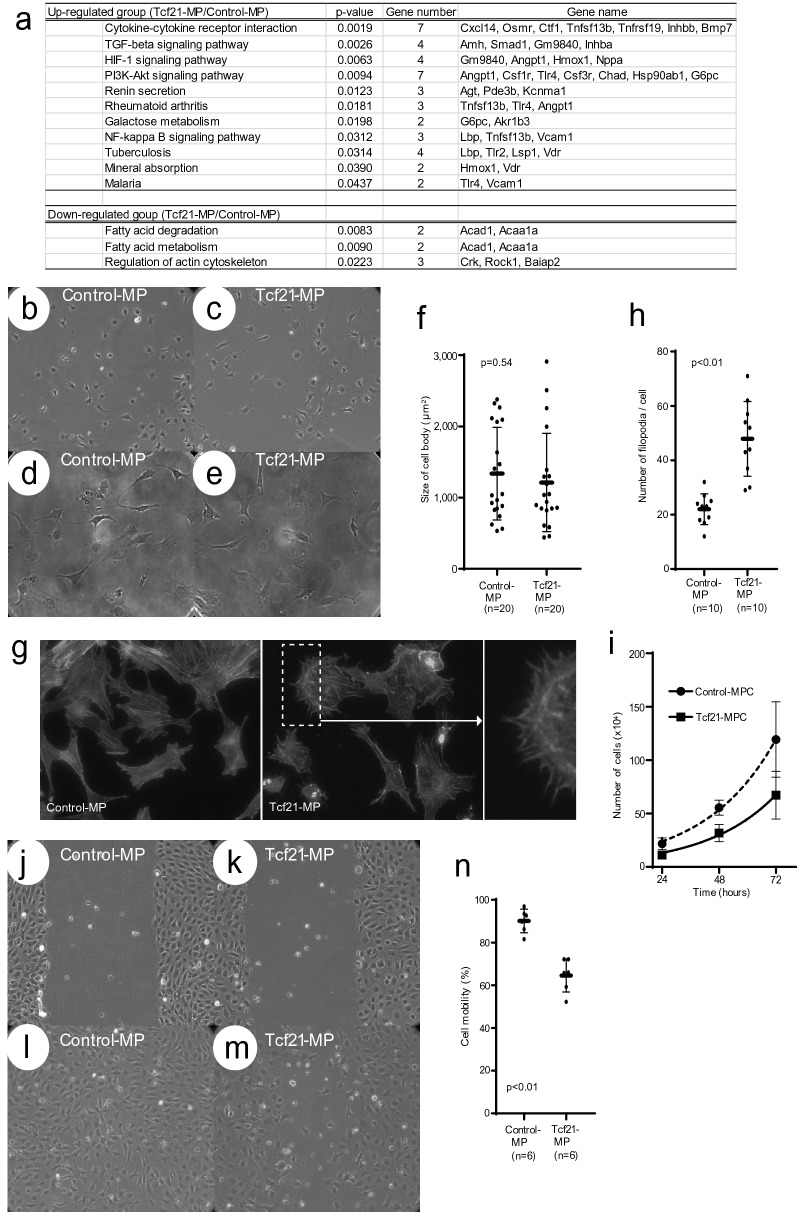
In vitro experiments using *Tcf21*-expressing murine podocyte cell line. **a** Based on DEGs of Tcf21-MPs/Control-MPs using microarray, the KEGG pathway frequency analysis presented up-regulated and down-regulated gene groups. **b**–**e**: Both cellular morphology of Control-MPs and Tcf21-MPs showed similar shape in low-power (**b**, **c**) and mid-power magnification (**d**, **e**). **f** The area dimension of both MPs was not statistically different (p = 0.54). **g** In high-power magnification, Tcf21-MP possessed rich actin-stained dendrites, filopodia. **h** The number of filopodia was significantly higher in Tcf21-MP than in Control-MP (p < 0.01). **i** Cell proliferation assay. Cell proliferation of Tcf21-MP was significantly lower than that of Control-MP at 24, 48 and 72 h (p = 0.04, 0.02, < 0.01, respectively). **j**–**m**: Wound assay for cell migration. The images showed at the experimental beginning (**j**, **k**) and 24hrs after the beginning (**l**, **m**). **n** The migration ability of TCF21-MP was significantly lower than that of Control-MP (p < 0.01). Statistical analysis was performed with Mann Whitney *U* test (**f**, **h**, **i**, **n**).

**Figure 4:**
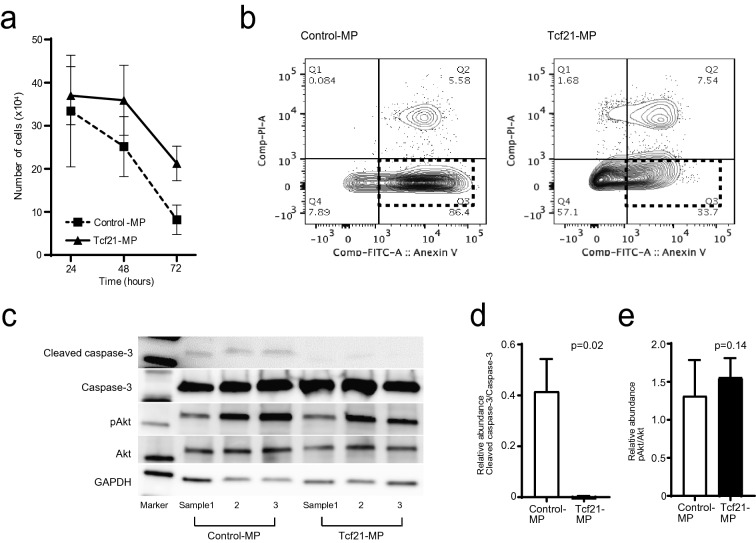
Anti-apoptotic effect of *Tcf21*-expressing murine podocyte cell line using ADR stimulation. **a** Tcf21-MPs could significantly survive at 48 (p = 0.04) and 72hrs (p < 0.01) after ADR treatments than Control-MPs, but not at 24hrs (p = 0.59). **b** Using annexin-V assay, apoptosis in Tcf21-MPs (scattered square in right) was remarkably suppressed than that of Control-MP (scattered square in left). **c** Western blot of cleaved caspase-3/caspase-3, pAkt/Akt and GAPDH, comparison between Control-MP and Tcf21-MP, treated with ADR. **d** The quantitative analysis of cleaved caspase-3/caspase-3 ratio. Tcf21-MP had less up-regulation of cleaved caspase-3 than Control-MP (p = 0.02). **e** The quantitative analysis of pAkt/Akt ratio. By contrast, pAkt/Akt ratio in Tcf21-MP wasn’t suppressed (p = 0.14). Statistical analysis was performed with Mann Whitney *U* test (**a**, **d**, **e**).

### Analysis of urinary TCF21 levels in human glomerular diseases

Here we analyzed urinary TCF21 concentration. In the analysis using total 156 urine samples, Urinary TCF21 levels showed a poor correlation with urinary protein levels overall (Fig. 2a, r 0.4639). Urinary TCF21 concentration was statistically significant among each glomerular disease, and showed a positive correlation among each glomerular disease (Fig. 2b, p < 0.01, with severity of proteinuria; 0.03 ± 0.03 g/gCre in healthy volunteers, 0.7 ± 0.5 g/gCre in NGD, 1.4 ± 1.5 g/gCre in IgAN, 6.2 ± 2.2 g/gCre in FSGS, 7.9 ± 3.2 g/gCre in MCD and 6.8 ± 4.6 g/gCre in MGN). To exclude the possibility that some factors such as microvilli or exosome from shed podocytes influence on the urinary TCF21 level, we compared TCF21 concentration of supernatants before and after the removal of microvilli or exosome by ultra-centrifugation in 3 patients with nephrotic syndrome. The changes in concentration was from 1,678.1 (before ultra-centrifugation) to 1,901.3 pg/mL (after ultra-centrifugation) in MGN patient No.1, from 1,612.1 to 2,547.7 pg/mL in MGN patient No.2, and from 3,320.5 to 2,925.6 pg/mL in MCD patient No.3. These results suggested that urinary TCF21 level was not influenced by factors derived from shed podocytes. Among 50 cases with available urine samples, a positive relationship between the histological TCF21 score and urinary TCF21 concentration was verified (Fig. 2c, p < 0.01). While in 19 cases with MGN, urinary TCF21 levels correlated with the strength of histological TCF21 expression (Fig. 2d, p = 0.01), but none was observed with anti-PLA2R positivity (Fig. 2e, p = 0.28). Finally, we investigated the changes in urinary TCF21 concentration in 29 cases with nephrotic syndrome depending based on treatment response variability. The downward rate of change was greater in the remission group (urinary protein < 0.3 g/gCre) than in non-remission group (Fig. 2f, p < 0.01). Here we show a case of steroid-sensitive MCD (Fig. 2g). At the time of treatment of this patient using glucocorticoid, urinary TCF21 concentration was continuously examined. During the process of remission, urinary TCF21 level decreased prior to complete recovery of urinary protein. Summarizing the above, urinary TCF21 concentration showed a positive relation with both urinary protein and podocyte expression level. Urinary TCF21 level was especially high in nephrotic syndrome but decreased following positive response to treatment.

### Microarray and KEGG pathway analysis of *Tcf21*-expressing murine podocyte cell line

Functional significance of *Tcf21* expression in podocyte was examined using the cultured murine podocyte (MP). We compared the differential mRNA expression between MPs with or without *Tcf21*-overexpression (Tcf21-MP or Control-MP) using cDNA microarray. First, we compared gene expression between Control-MP and Tcf21-MP, focusing podocyte-specific molecules. Fifty-three podocyte-expressing genes were selected among 8 × 60 K probes on non-filtering database. However, all individual gene expression excluding *Tcf21* was statistically significant between both cell lines (Supplementary Table 1). Next, after scaling and filtering probe signals, cDNA microarray analysis identified DEGs associated with *Tcf21*-overexpression in MP. In Tcf21-MP/Control-MP, 180 up-regulated genes were identified as DEGs, and 51 genes of them were twofold up-regulated. By contrast, 77 down-regulated genes were identified, and 6 genes of them were half folds down-regulated. The KEGG pathway frequency analysis presented that up-regulated gene groups (Tcf21-MP/Control-MP) included cytokine-cytokine receptor interaction, TGF-beta signaling pathway, HIF-1 signaling pathway, PI3K-Akt signaling pathway, renin secretion, rheumatoid arthritis, galactose metabolism, NF-kappa B signaling pathway, tuberculosis, mineral absorption, and malaria (Fig. 3a). By contrast, down-regulated gene groups included fatty acid degradation, fatty acid metabolism and regulation of actin cytoskeleton. Therefore, we confirmed some cellular signaling pathway influenced by *Tcf21*-overexpression in podocytes.

### Morphological and cellular functional analysis of *Tcf21*-expressing murine podocyte cell line

Based on the results of microarray and KEGG pathway analysis, including cytokine-cytokine receptor interaction, TGF-beta signaling pathway, PI3K-Akt signaling pathway and regulation of actin cytoskeleton, we examined morphology and cellular function of Tcf21-MP. Low power field micrograph yielded no difference in morphology including primary processes of Tcf21-MP and Control-MP (Fig. 3b–e), either in cell size (Fig. 3f, p = 0.54). As for high power field micrograph, the number of actin-stained filopodia was significantly higher in Tcf21-MP than in Control-MP (Fig. 3g, h, p < 0.01). Next, we focused on cell migration and proliferation, and investigated both assays. Cell proliferation of Tcf21-MP was lower than that of Control-MP throughout the examining time course (Fig. 3i, p = 0.04 at 24 h, 0.02 at 48 h, < 0.01 at 72 h, respectively). And we used the wound assay for cell migration. Tcf21-MP had less migration potency than Control-MP (Fig. 3j–m). The migration ability of Tcf21-MP was significantly lower than that of Control-MP (Fig. 3n, p < 0.01). Finally, as a verification method, we investigated 2 genes expression related with actin cytoskeleton regulation in KEGG pathway analysis, including *Rock1* and *Baiap2*, by using in vitro injured podocytes. We used an ADR-induced in vitro model of podocyte injury mimicking nephrotic syndrome. Inducing cell death of the cultured murine podocytes by adding ADR, this in vitro model precisely described the progression of podocyte injury in nephrotic syndrome. In detail, we compared *Rock1* and *Baiap2* mRNA expression between Tcf21-MP versus Control-MP under ADR treatment. We firstly detected the constitutional expression of *Tcf21* in Tcf21-MPs with up-regulation trend (Supplementary Fig. 6a, p = 0.01). As a result, both *Rock1* and *Baiap2* mRNA expression of Control-MPs significantly decreased after ADR treatment (Supplementary Fig. 6b, c, p = 0.03). Moreover, the difference of both *Rock1* and *Baiap2* between Tcf21-MPs and Control-MPs at pre-treatment permissive condition disappeared at 6 (p = 0.4, > 0.9, respectively) and 12 h (p = 0.4, 0.2, respectively) after ADR treatment. Although the cause was unknown, there was no difference in gene expression related with actin cytoskeleton after ADR treatment using in vitro model of injured podocyte. These results convinced us that *Tcf21* act some molecular expression associated with the organization of actin cytoskeleton. In conclusion, the in vitro experiments and gene expression data revealed that *Tcf21*-overexpression promoted filopodia formation composed of actin filament-rich and also that it affected cell proliferation and migration.

### Anti-apoptotic effect of *Tcf21*-expressing murine podocyte cell line

Finally, we examined processes of cellular survival because microarray pathway analysis showed upregulation of cellular survival-related signaling pathways including TGF-beta signaling pathway, PI3K-Akt signaling pathway and NF-kappa B signaling pathway as mentioned above. We also used an ADR-induced in vitro model of podocyte injury mimicking nephrotic syndrome. First, Tcf21-MPs could significantly survive at 48 (Fig. 4a, p = 0.04) and 72hrs (p < 0.01) after ADR treatments than Control-MPs, but not at 24hrs (p = 0.59). Using fluorescence-activated cell sorting with annexin-V, apoptosis in Tcf21-MPs was remarkably suppressed compared to that in Control-MP (Fig. 4b). We examined by western blot to check the regulation of apoptosis-related signaling pathway including caspase-3 and Akt signaling. Western blot analysis of cleaved caspase-3 expression confirmed that Tcf21-MP had less upregulation of cleaved caspase-3 than Control-MP (Fig. 4c, d, raw gel images in Supplementary Fig. 5a–c, p = 0.02). By contrast, pAkt/Akt expression ratio was not influenced in Tcf21-MP, so PI3K-Akt signaling pathway was unrelated with *Tcf21*-overexpression (Fig. 4c, e, raw gel images in Supplementary Fig. 5c–e, p = 0.14). This indicates that *Tcf21* expression suppressed apoptosis in injured podocyte via caspase-3 signaling pathway, leading a conclusion that *Tcf21* expression had a significant impact on podocyte cell-survival function.

**Figure 5:**
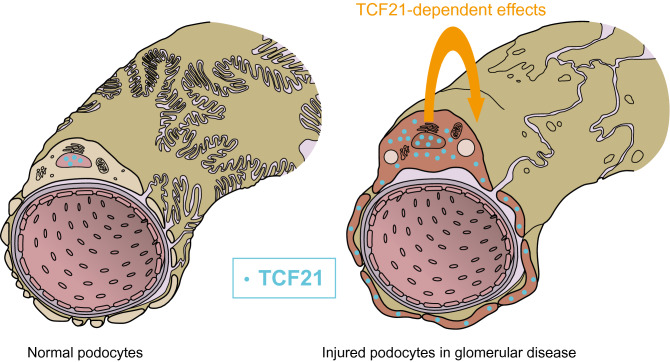
Mechanism hypothesis of TCF21 expression in injured podocytes of glomerular disease. TCF21 expresses in nucleus of normal podocytes on glomerular capillary (left). By contrast, TCF21 highly expresses in both nucleus and cytoplasm of injured podocytes on glomerular disease including nephrotic syndrome (right). Therefore, TCF21 overexpression may have cellular functional effect in injured podocyte. This image was drawn by MEDICAL EDUCATION INC. (Tokyo, Japan), and all copyright was assigned to corresponding author, J.U.

## Discussion

TCF21 is a key transcription factor in nephrogenesis, especially in glomerular maturation^10,12^. However, little is known about the contribution of TCF21 to glomerular injury. Focusing on its significance, we proved podocyte-expressed TCF21 in various glomerular diseases using kidney tissue from both humans and rat models. Also, we sought to establish the hypothesis that urinary TCF21 would be a useful biomarker related with kidney injuries. In vitro analysis suggested that an increase of *Tcf21* expression in podocytes mediates the anti-apoptosis function and cytoskeletal changes in injured podocytes, which supports the hypothesis that an increase of *Tcf21* expression plays a role in protecting glomerular function (Fig. 5). Given these findings, we believed that podocyte-expressed TCF21 in various glomerular diseases has an impact on determining the podocyte injury phenotypes. Since most of the previously reported podocyte-specific molecules were causal factors of podocyte injury such as hereditary or familial FSGS^13^, our work on the recognition of TCF21 as a podocyte-protective molecule may be especially useful for further studies.

Focusing on the influence of TCF21 on kidney diseases, we observed differences between previously published reports and the present study. Even though diabetic nephropathy was not examined in our study, a previous gene-expression study using a mouse diabetic kidney disease (DKD) model highlighted significant changes in *Tcf21* expression^14^. Podocyte-specific genes expression including *Tcf21* showed an increase in an early-stage DKD model, whereas they decreased in a chronic-stage model. This stage-dependent variation in *Tcf21* expression can be explained by the severity of the podocyte injury. Podocyte injury ultimately results in podocyte loss, leading to a decrease in *Tcf21* expression. In our study, TCF21 was not expressed in glomerular segmental sclerotic legions (data not shown), and the TCF21 expression score was markedly lower in human FSGS than in other forms of nephrotic syndrome including MCD and MGN. The previous study provided insight into this mechanism, suggesting that the lower score in FSGS could be explained by the podocyte injury and podocyte loss.

The results of our present study led us to suppose that urinary TCF21 is derived from podocytes. Two points should be mentioned to explain this mechanism: the reason why this intranuclear factor was found in urine, and whether or not urinary TCF21 was from shed podocytes. As for the first question, even though TCF21 has been known to show intranuclear expression as a bHLH transcription factor, it is also found in the cytoplasm^15^. In podocyte, also, TCF21 was detected in the podocyte cytoplasm in injured podocytes. In a previous report, Zhou et al. described that one of the transcription factors, WT1, could be found in urinary exosomes^16^. They proved that exosomes contained cytosolic proteins existing in the nucleus and the cytosol by detecting urinary exosomal WT1 in rats and mice models of FSGS. This supported the hypothesis that transcription factor exists not only in the nuclear but also in the cytoplasm of injured podocytes. As for the second question about the influence of shed podocytes, even though podocyte microvilli or exosome are known to be shed into the urine in nephrotic syndrome^17^, we did not assume that urinary TCF21 in our experiments were derived from shed podocytes. The reason was that we measured urinary TCF21 level in supernatants after the removal of microvilli or exosome by ultra-centrifugation and confirmed that microvilli or exosome from shed podocytes did not influence on urinary TCF21 level. This process excluded the possibility that urinary TCF21 of shed podocytes increased the level of urinary TCF21. It led us to suppose that the increase in urinary TCF21 in glomerular diseases resulted from some factors other than podocyte shedding. The detailed mechanisms remain to be elucidated, but we hypothesized that urinary TCF21 might derive from podocytes after cell membrane damage. In MGN, as an example of this mechanism, the complement pathway is activated on the podocyte cell membrane, leading to direct damage to the podocyte membrane by a membrane attack complex of complement (C5b-9)^18^. This suggests that podocyte membrane injury may be crucial in severe nephrotic syndrome. Thus, both the increase of TCF21 expression of injured kidney segments including podocytes and its appearance in the urine may lead to urinary TCF21 elevation in various kidney diseases. Our study has the following limitations. Since the molecular weight of TCF21 (20,000) is significantly lower than that of albumin (68,000), the charge specificity and interaction of TCF21 with other proteins including albumin remain unclear. Our study therefore did not preclude the possibility that TCF21 was filtered across the capillaries of the glomerulus. The same can be said of TCF21 secretion and reabsorption failure in the renal tubules. Although we confirmed rare TCF21 expression in tubular cells using immunohistochemistry and mRNA expression analysis, the possibility of TCF21 secretion and reabsorption failure in the renal tubules cannot be completely denied.

Finally, the role of TCF21 in injured podocytes should be described. Maezawa et al*.* previously reported *Tcf21* knockout adult mice showed the development of FSGS with age, and the DKD models changed for the worse^11^. This finding confirmed the protective effect of *Tcf21* on podocytes in a mouse model in vivo. Through this comprehensive genetic testing, angiogenic factors including vascular endothelial growth factor (*Vegfa*) were downregulated in *Tcf21* knockout mice. This led us to the hypothesis that *Tcf21* acts upstream of the angiogenic network. In our study in vitro, even though no interaction existed between *Tcf21* and the angiogenic network, we pointed out two factors that regulated podocyte injury. One is the prevention of programmed cell death by the anti-apoptosis function and the other is the inhibition of cell migration transmitted through actin cytoskeleton reorganization. Podocyte apoptosis has been considered to be important as a final common pathway in nephrotic syndrome represented by FSGS^19,20^. In podocyte injury, cell death including apoptosis is initiated by several types of apoptosis-inducing intracellular signal transmissions such as the caspase and PI3K-Akt pathway^19–22^. The renin-angiotensin system inhibitor, statin, and vitamin D with their anti-apoptotic function are known to be protective reagents for injured podocytes^21,23,24^. *Tcf21* acting as an anti-apoptotic player can help stop podocytes from resulting in cell death. Second, actin cytoskeleton reorganization is well documented in podocyte injury^25,26^. Podocytes are firmly attached to the glomerular basement membrane and resist strong filtration pressure from the basement membrane. The foot process is also equipped with various components of actin cytoskeleton. In nephrotic syndrome or FSGS, the actin cytoskeleton dysregulation is dynamically reorganized along with foot process effacement and detachment from basement membrane by loss of adhesion. According to the present microarray and KEGG pathway analysis, two down-regulated genes are associated with actin organization. Rho-associated coiled coli-containing protein kinase 1 (ROCK1) was previously reported as a key molecule of podocyte-specific kidney diseases^25^. ROCK including two isoforms, ROCK1 and ROCK2, is a downstream effector kinase of RhoA to mediate the effects of RhoA on the formation of stress fiber and focal contact by phosphorylating myosin light chain, which binds by myosin II and thus increase in contractility. A previous reports mentioned that non-selective ROCK inhibitor in PAN experimental models ameliorated proteinuria^27^. Moreover ROCK1-deficient mice with diabetes less develop diabetic nephropathy, and podocyte-specific constitutive active ROCK1 murine model deteriorated kidney dysfunction^28^. Alternatively, brain-specific angiogenesis inhibitor 1-associated protein 2 (BAIAP2, also known as insulin receptor substrate 53, IRSp53) was previously reported to the relationship with a proteinuric signaling pathway of podocytes^29^. The Rho family GTPase Cdc42 is a key player for synaptopodin-associated podocyte actin dynamics, and BAIAP2 is one of downstream effector proteins in the filopodia formation initiated by Cdc42. So synaptopodin suppresses Cdc42-BAIAP2 signaling pathway for anti-proteinuric function of podocytes. Therefore, ROCK1 and BAIAP2 down-regulation in podocytes can directly protect from podocyte dysfunction via actin cytoskeleton organization. In FSGS, as a result of actin cytoskeleton reorganization, podocytes easily lose the contact with a fixed position of basement membrane, detaching from the basement membrane or forming a bridge with the Bowman's capsule. In our experiment, Tcf21-MPs showed low moving activity, so we may expect that *Tcf21* forces injured podocytes to keep locating at fixed position along the capillary walls in nephrotic syndrome or FSGS. *Tcf21* therefore affects the reorganization of the actin cytoskeleton, maintains the architecture of the foot process, and thus acts as an anti-proteinuric factor. In addition, it helps prevent the formation of glomerular sclerotic lesions by inhibiting the mobility of podocytes and their detachment from the basement membrane. Acting upstream of both of these pathways as a transcription factor, *Tcf21* would be essential in protecting injured podocytes.

Based on this study, we demonstrated the transition in podocyte TCF21-expression of glomerular diseases and showed that it may function as a urinary biomarker in various kidney injuries. Our study also illustrated its in vitro functional significance as a podocyte protective factor. Since most of the previously found podocyte-specific molecules act like causal factors of podocyte injury, identifying TCF21 as a podocyte protective molecule is especially meaningful. We expect that the elucidation of this mechanism, which is involved in protecting podocytes, will help accelerate the development of drugs that can inhibit the formation of FGS lesions, which are known as a final common pathway in most forms of glomerular injury.

## Materials and methods

### Human samples

We selected human kidney samples from 62 adult cases of glomerular diseases histologically diagnosed at University of Tsukuba Hospital. The glomerular diseases included 21 samples of immunoglobulin A nephropathy (IgAN: mean age 36.5 ± 13.0yo, male/female 10/11), 3 samples of focal segmental glomerulosclerosis (FSGS: mean age 29.7 ± 13.3yo, male/female 2/1), 6 samples of minimal change disease (MCD: mean age 55.0 ± 13.6yo, male/female 3/3) and 23 samples of membranous glomerulonephritis (MGN: mean age 62.6 ± 14.7yo, male/female 16/7). All patients with both FSGS and MCD, and 14 cases of MGN clinically presented with nephrotic syndrome. In addition, we prepared 9 non-glomerular disease (NGD: average age 51.9 ± 15.1yo, male/female 8/1) samples from cases manifesting tubulo-interstitial disease and minor histologically diagnosed glomerular abnormalities as controls.

We collected human 156 urine samples (mean age 51.4 ± 17.9yo, male/female 81/75). Of these, the urine samples included 7 healthy volunteers (mean age 43.7 ± 11.8yo, male/female 3/4), 6 cases of NGD (mean age 56.2 ± 15.0yo, male/female 6/0), 46 of IgAN (mean age 37.4 ± 16.4yo, male/female 25/21), 8 of FSGS (mean age 38.5 ± 23.4yo, male/female 5/3), 7 of MCD (mean age 57.0 ± 13.5yo, male/female 2/5) and 32 of MGN (mean age 62.6 ± 14.2yo, male/female 19/13). The spot urine samples were used for routine analysis, and 24-h pooled urine was used to investigate time-dependent change.

### Animals

Male SD rats were purchased from SLC Japan (Shizuoka, Japan). Puromycin aminonuclease (PAN)-peritoneally injected rats at 7 weeks after birth were prepared as a nephrotic model. Control rats were treated with saline solution. At 10 days (the acme of the nephrotic phase) and 28 days (the recovery phase) after PAN treatment, the animals were sacrificed, and their kidneys were removed and formalin-fixed for embedding in paraffin sections. More than three samples were analyzed in each group.

### Histological analysis

Paraffin sections were stained with hematoxylin–eosin and periodic acid-Schiff for light microscopy using the immunohistochemical technique. Paraffin sections were deparaffinized with xylene and hydrated with graded ethanols. A microwave oven was used for antigen retrieval. Briefly, each section was incubated with primary antibody overnight at 4 degrees C and with secondary antibody for 1 h at room temperature. The primary antibodies were polyclonal anti-TCF21 antibody (Lot Number A75217, pAb; rabbit IgG: 1:250 dilution; Sigma-Aldrich, MO, USA), monoclonal anti-synaptopodin Ab (mAb; mouse IgG, 1:5 dilution; Progen Biotechnik, Germany), and anti-nephrin pAb (sheep IgG: 1:200 dilution; R&D systems, MN, USA). Anti-TCF21 antibody was listed in the human protein atlas (https://www.proteinatlas.org/ENSG00000118526-TCF21/antibody). The specificity of anti-TCF21 antibody was fully identified because definite reaction was confirmed by western blot. Also it was previously used in chromatin immunoprecipitation of human cultured cell^30^. The secondary antibodies and detection used for immunohistochemical studies were: peroxidase-conjugated anti-rabbit (for TCF21), anti-mouse IgG pAb (for synaptopodin, Nichirei Bioscience, Tokyo, Japan), peroxidase-conjugated anti-gout/sheep IgG mAb (for nephrin, Sigma-Aldrich), and biotin-conjugated anti-rabbit IgG pAb (for TCF21, Nichirei Bioscience) with alkaline phosphatase-conjugated streptavidin. The detection kits were the DAB detection kit (Dako, Denmark) and alkaline phosphatase substrate kit IV (Vector Laboratories, CA, USA). In the single immunohistochemistry for anti-TCF21, the sections were nuclear stained using hematoxylin. To verify the specificity of polyclonal anti-TCF21 antibody, we stained with the antibody supernatant reacted with recombinant human TCF21 peptide antigen that was a full length protein (Abcam, ab152730, Cambridge, UK). The applied solution was made on 1 mol/20 mol of antibody/antigen peptide ratio, reacted overnight at 4 degrees C, then centrifuge.

The scoring of each section was investigated by a semi-quantitative method. Five glomeruli were selected in a random manner in each section, and the different staining patterns of each glomerulus were then evaluated and compared with representative glomeruli in the NGD sample. The scoring was done as follows by two expert renal pathologists, belonging to the Renal Pathology Society: 0, the nuclear expression of all podocytes did not increase; 1, the nuclear expression of segmental podocytes (less than 50%) increased; 2, the nuclear expression of global podocytes (50% or more) increased; 3, the cytoplasmic expression of global podocytes in diffuse glomeruli (50% or more) was seen; 4, the cytoplasmic expression of global podocytes in most glomeruli (90% or more) was seen. For PAN rat experiments, the single immunohistochemistry for anti-TCF21 without nuclear stain and semi-quantitative analysis same as human materials were used.

Anti-phospholipase A2 receptor (PLA2R) staining on the paraffin sections of MGN cases was examined as previously described^31^. The antibodies were anti-PLA2R1 polyclonal antibody (pAb; rabbit IgG: 1:50 dilution; Sigma-Aldrich) and Alexa488-conjugated anti-rabbit IgG antibody (1:1,000 dilution, Thermo Fisher Scientific Inc., MA, USA). Alexa488 fluorescence was observed by fluorescence microscopy.

### Web-based mRNA expression database

To support the quantification of gene expression in human kidney, we used web-based mRNA expression database, Nephroseq datasets (www.nephroseq.org, < March, 2020 > , University of Michigan, Ann Arbor, MI). In detail, we compared human *TCF21* mRNA expression between glomeruli and tubulointerstitium in normal human renal tissue, and among some kidney diseases including healthy living donor, MN and MCD.

### Measurement of TCF21 concentration

The TCF21 concentration was measured in urine and serum samples using a TCF21 enzyme-linked immunosorbent assay kit (ELISA, Cusabio Biotech, China). The procedure was the standard sandwich method under a products-adjunctive protocol. Absorbance determination with a 3,3′,5,5′-tetramethylbenzidine substrate was determined using the MTP-300 microplate reader (Corona Electric Co., Ltd., Ibaraki, Japan). The urinary TCF21 concentration was corrected using the urinary creatinine (Cre) concentration, whose unit was titer pg/mg Cre.

### Culture of *Tcf21*-expressing murine podocyte cell line

An immortalized murine podocyte cell line was provided with Dr. Mundel’s permission (Harvard University, USA) and treated as previously described^32^. As briefly mentioned, podocytes could be maintained in a permissive condition using RPMI 1,640 medium (Sigma-Aldrich) with 10% fetal bovine serum (FBS, Sigma-Aldrich), 10 units/mL recombinant murine interferon-gamma (PeproTech, NJ, USA), penicillin and streptomycin at 33 degrees C, and 5% CO_2_ in non-coated wells. For differentiation, the podocyte cell line in permissive condition was converted to a non-permissive condition without interferon-gamma at 37 degrees C and 5% CO_2_ for 7 days, in type-I-collagen (Nitta Gelatin Inc., Osaka, Japan)-coated wells. The *Tcf21*-expressing murine podocyte cell line in permissive condition was produced using lentiviral infection by a commercial viral vector supplier (SIRION Biotech GmBH, Martinsried, Germany). To set up the lentiviral transduction into the murine podocyte cell line, they investigated whether CMV, EF1a, or Ubic promoter induced the highest expression of CopGFP. As a result, lentiviral pCLV-CMV-*Tcf21*-IRES-CopGFP-T2a-Puro vector was transduced into a podocyte cell line to express murine *Tcf21*, and viral vector-transducing cells were selected using puromycin (cell line name: Tcf21-MP). As a control cell line, the same lentiviral vector without *Tcf21* cDNA was induced in a murine podocyte cell line (cell line name: Control-MP). The induction of *Tcf21* expression was confirmed by reverse transcription-polymerase chain reaction (RT-PCR), quantitative PCR of *Tcf21* (by SIRION Biotech GmBH and us), microarray (by us), immunoblot of TCF21 (by SIRION Biotech GmBH), immunocytochemistry of TCF21 (by us).

### RNA extraction, microarray, KEGG pathway analysis and quantitative Real-Time PCR

Total RNA was extracted with the acid guanidinium thiocyanate-phenol–chloroform extraction method, using ISOGEN (Nippon Gene Co., Ltd., Tokyo, Japan). Triplicate samples of Tcf21-MP and Control-MP in non-permissive condition were prepared for microarray analysis. SurePrint G3 Mouse GE 8 × 60 K Microarray (Agilent Technologies Inc., CA, USA) was used for the microarray analysis, which was performed and analyzed by CDM Center, Takara Bio Co. (Mie, Japan). After the signal filtering, each signal probe between Tcf21-MPs vs Control-MPs was compared by t-test, then false discovery rate (FDR) was calculated by Benjamini–Hochberg method. Among signal probe of FDR ≤ 0.05, up-regulated and down-regulated signal probes were selected as differentially expressed genes (DEGs). Finally, on the basis of DEGs data, the pathway analysis using the Kyoto Encyclopedia of Genes and Genomes (KEGG) pathway database (https://www.genome.jp/kegg/) was added to elucidate the trivial association with the molecular network, which was analyzed by SCM Center, Takara Bio Co. (Shiga, Japan). As a verification method, to compare mRNA expression between Tcf21-MPs vs Control-MPs in permissive condition, the quantitative Real-Time PCR (qPCR) was done using Applied Biosystems TaqMan Assays with standard protocol (Thermo Fisher Scientific). Murine *Tcf21* (Mm00448961_m1), *Rock1*(Mn00485745_m1), *Baiap2* (Mn01241971_m1) and *Gapdh* (Mn99999915_g1) were used as the probes for TaqMan Assays. Relative quantitative expression was assessed with delta Ct compared to *Gapdh* expression. Triplicate samples of Tcf21-MP and Control-MP in permissive condition were prepared for qPCR analysis.

### Assay for morphology and cellular function

Tcf21-MP and Control-MP were assessed and compared by their association with cellular morphology and cellular function. To measure the cellular size, podocytes in non-permissive condition were photographed in a random manner, and the image was measured by the number of pixels. The cell proliferation assay was performed as follows: 9.5 × 10^4^ cells of Tcf21-MP and Control-MP in permissive condition were plated in the wells of six-well culture plates, and the numbers of podocytes were then counted at 72 h. To assess the cell motility, a wound-healing assay was performed as previously described^33^. Podocytes in non-permissive condition were grown to a confluent monolayer, and a wound was introduced using a 200-uL pipette tip. After 24 h, the images were obtained in a random manner, and we counted the cell numbers within the wound.

### Immunocytochemical analysis for actin cytoskeleton

To identify changes in the organization of actin cytoskeleton, after the incubation of podocytes, cells were washed three times with PBS, fixed with 4% paraformaldehyde, permeabilized with 0.3% Triton-X 100, incubated with 5% normal donkey serum (Sigma-Aldrich), then stained with Acti-stain^TM^555 Fluorescent Phalloidin for TRITC excitation filter setting (1:150 dilution; Cytoskeleton, Inc., CO, USA) and 4′,6-diamidino-2-phenylindole for the detection of actin cytoskeleton and nuclear DNA. Podocytes in permissive condition were observed as staining patterns and photographs were taken using fluorescent microscopy in a random manner, and the number of actin-stained filopodia per each cell were counted. Ten cells of each Tcf21-MP and Control-MP were analyzed, and the average number of filopodia between both cell lines was compared.

### Podocyte apoptosis induced by adriamycin in vitro

As an in vitro nephrosis model, podocytes were incubated with adriamycin (ADR) as previously described^34^. Cell apoptosis was induced in podocytes in non-permissive condition with the stimulation of ADR. In the stimulating condition, the concentrations of ADR in culture medium was 1ug/uL, and the observation time was 72hrs. To assess the degree of apoptosis, an annexin V-FITC kit (Beckman Coulter Inc., France) and fluorescence-activated cell sorting were used as user’s manual. Cultured cells stimulated by 1ug/uL ADR for 24hrs were suspended in PBS containing 3% FBS, then stained with FITC-conjugated annexin V. The analysis was performed by BD FACS Canto II (BD Biosciences, NJ, USA), and we observed annexin V-positive, propidium iodide-negative cells as the apoptotic cells. We confirmed that transfected GFP luminescence of both cell lines was ignorable comparing with the luminescence of FITC-annexin V.

Additionally, caspase-3 and phosphoinositide 3 kinase (PI3K)-dependent Akt signaling activation was investigated with immunoblotting. Podocytes stimulated by 1 ug/uL Dox for 12hrs underwent immunoblotting as follows. The primary antibodies were anti-cleaved caspase-3 pAb (1:1,000 dilution; rabbit Ab, R&D systems), anti-caspase-3 pAb, (1:1,000 dilution; rabbit IgG, Cell Signaling Technology, MA, USA), anti-pAkt (Ser473) Ab (1:2,000 dilution, Cell Signaling Technology), anti-Akt Ab (1:1,000 dilution, Cell Signaling Technology) and anti-GAPDH pAb (1:25,000 dilution; rabbit IgG, Cell Signaling Technology). The secondary antibodies and detection reagents were HRP-conjugated anti-rabbit IgG (1:2,000 dilution; Cell Signaling Technology), StrepTactin HRP-Conjugate (Bio-Rad, CA, USA), and Amersham ECL prime western blotting detection reagent (GE Healthcare UK Ltd., Buckinghamshire UK). Detection and quantification were performed with ImageQuant LAS 4,000 (GE Healthcare UK Ltd.). The cleaved caspase-3/caspase-3 and pAkt/Akt expression ratio was calculated and compared between Tcf21-MP and Control-MP.

### Statistical analysis

All cell culture experiments were performed using at least triplicate samples. The quantitative data were written as mean value ± standard deviation (SD) in each figure. To analyze the parameters, the quantitative variables between two groups were compared with Mann Whitney U test. The quantitative variables among three or more groups were compared with the Kruskal–Wallis test. The correlation coefficient was judged with Spearman’s rank correlation coefficient. Statistical significance was established at p < 0.05. The IBM SPSS Statistics for Windows version 25.0 (IBM Corp., Armonk, NY, USA) and GraphPad Prism version 7.00 for Windows (GraphPad Software, CA, USA) was used for all of the statistical analysis and production of figures.

### Study approval

Human: This research protocol was approved by the Ethics Committee of University of Tsukuba Hospital (H20-273, H26-26). Additionally, the comprehensive permitting system for the treatment of human biological samples at University of Tsukuba Hospital was utilized as a foundation for this research protocol (H26-26). Written Informed consent was obtained from all participants. Additionally, an announcement of this study was simultaneously posted at the outpatient clinic of our institute. All experiments were conducted according to the Declaration of Helsinki. The medical records of the patients were anonymized for personal information so that they cannot be identified. All methods were performed in accordance with ethical guidelines for epidemiology research in Japan and use guidelines and regulations of the University of Tsukuba.

Animal: This research protocol was approved by the Laboratory Animal Resource Center at the University of Tsukuba (H25-59, H26-26). All methods were performed in accordance with the animal care and use guidelines and regulations of the University of Tsukuba.

## Supplementary information

Supplementary Table 1 Comparison of cDNA microarray data between Control-MP and Tcf21-MP, focusing podocyte-expressing molecules. Fifty-three podocyte-expressing genes were selected among 8x60K probes. However, all individual gene expression excluding Tcf21 was statistically significant between both cell lines. (PDF 96 kb)

Supplementary Figure 1 The specificity of polyclonal anti-TCF21 antibody. To verify the specificity of polyclonal anti-TCF21 antibody, we stained with the antibody supernatant reacted with recombinant human TCF21 peptide antigen. a: Reaction with anti-TCF21 antibody in the MGN sample. b: Negative control, immunohistochemistry without primary antibody reaction on the serial section. c: Antibody-absorption test using recombinant TCF21 peptide antigen. In the section using supernatant absorbed by TCF21 antigen, the stain was not found, comparing to positive stain with no-absorbing primary antibody (a). (PDF 889 kb)

Supplementary Figure 2 Histological analysis of TCF21 in human kidney biopsy samples with low power-magnification view. a: In NGD sample, TCF21 was mainly and weakly expressed in podocytes of glomeruli. By contrast, TCF21 expression was rarely seen in the tubulointerstitial area. The positive reaction of tubular cells was unremarkable. b: In MGN sample, TCF21 was also mainly expressed in podocytes of glomeruli, comparing to tubulointerstitial cells. (PDF 429 kb)

Supplementary Figure 3 TCF21 expression data from Nephroseq datasets (Nephroseq.org). a: TCF21 expression in normal human renal tissues, comparison between glomeruli and tubulointerstitium (n=12, Fold Change:-62.414, p=8.48E-12; Lindenmeyer Normal Tissue Panel, Nephroseq.org). Sample size (n) is indicated in parenthesis. b: TCF21 expression in Healthy Living Donor (n=21), Membranous Glomerulonephropathy (n=21) and Minimal Change Disease (n=14) (modified from Ju CKD Glom, Nephroseq.org). (PDF 131 kb)

Supplementary Figure 4 Histological analysis of TCF21 in rat nephrosis model. a, b: TCF21 stain in the nephrotic phase (day 10, a: PAN nephrosis model, b: control rat). c, d: The TCF21 stain in the recovery phase (day 28, c: PAN nephrosis model, d: control rat). The TCF21 in the nephrotic phase (a) highly expressed than that in control one (b), and then the TCF21 expression returned to weak level in the recovery phase (c, d). (PDF 267 kb)

Supplementary Figure 5 Raw gel images of Figure 4c. Western blot of cleaved caspase-3 (a), caspase-3 (b), GAPDH (c), pAkt (d), Akt (d). (PDF 520 kb)

Supplementary Figure 6 Comparison of Tcf21 (a), Rock1 (b) and Baiap2 (c) mRNA expression between Tcf21-MPs versus Control-MPs treated with ADR. a: Tcf21 constantly expressed in Tcf21-MPs with up-regulation trend (p=0.01). b: Rock1 expression of Control-MPs significantly decreased after ADR treatment (p=0.03). The difference of Rock1 between Tcf21-MPs and Control-MPs at pre-treatment permissive condition disappeared at 6 and 12 hours after ADR treatment (p=0.4, 0.4, respectively). C: Baiap2 expression of both MPs significantly decreased after ADR treatment (p<0.01, <0.01, respectively). The difference of Baiap2 between Tcf21-MPs and Control-MPs at pre-treatment permissive condition disappeared at 6 and 12 hours after ADR treatment (p>0.9, 0.2, respectively). Statistical analysis was performed with the Kruskal-Wallis test or Mann Whitney U test. (PDF 76 kb)
