# Supplementary material for: Transcription factor 21 expression in injured podocytes of glomerular diseases
Source: Sci Rep. 2020 Jul 13;10:11516. doi: 10.1038/s41598-020-68422-3 (PMC7359327; doi:10.1038/s41598-020-68422-3)
Supplement: Supplementary file 1 — Comparison of cDNA microarray data between Control-MP and Tcf21-MP, focusing podocyte-expressing molecules. Fifty-three podocyte-expressing genes were selected among 8x60K probes. However, all individual gene expression excluding Tcf21 was statistically significant between both cell lines. (PDF 96 kb) [file 41598_2020_68422_MOESM1_ESM.pdf]

Transcription factor 21 expression in injured podocytes of glomerular diseases

Joichi Usui[1,3], Misa Yaguchi[1], Satoshi Yamazaki[2,4], Mayumi Takahashi-Kobayashi[1], Tetsuya Kawamura[1], Shuzo Kaneko[1], Surya V Seshan[3], Pierre Ronco[5], Kunihiro Yamagata[1]

[1]Department of Nephrology and [2]Division of Stem Cell Therapy, Faculty of Medicine, University of Tsukuba, Tsukuba. Ibaraki, 305-8576, Japan.

[3]Department of Pathology and Laboratory Medicine, Weill Cornell Medicine, NY, NY, 10065, USA.

[4]Devision of Stem Cell Therapy, Distinguished Professor Units, The Institute of Medical Science, The University Tokyo, Tokyo, 108-8639, Japan.

[5]SorbonneUniversité, Université Pierre et Marie Curie Paris 06, and Institut National de la Santé et de la Recherche Médicale, Unité Mixte de Recherche S1155, Paris, France.

*Corresponding author: Joichi Usui, M.D., Ph.D.

Department of Nephrology, Faculty of Medicine, University of Tsukuba

1-1-1 Tennodai, Tsukuba, Ibaraki 3058575, Japan

TEL&FAX: +81-29-853-3202

E-MAIL: j-usui@md.tsukuba.ac.jp

Group	Gene symbol	Log ratio	Control MP-gsignal average	Tcf21 MP-gsignal average	Description
Transcription factor	*Tcf21*	2.117629368	13.33451639	57.86916675	Mus musculus transcription factor 21 (Tcf21), mRNA [NM_011545]
	Wt1	-0.211402355	456.2573985	394.0682723	Mus musculus Wilms tumor 1 homolog (Wt1), mRNA [NM_144783]
	Lmx1b	-0.248036568	107.7057112	90.69269047	Mus musculus LIM homeobox transcription factor 1 beta (Lmx1b), mRNA [NM_010725]
	Smarcal1	-0.032553693	1582.926796	1547.608808	Mus musculus SWI/SNF related matrix associated, actin dependent regulator of chromatin, subfamily a-like 1 (Smarcal1), mRNA [NM_018817]
	Mafb	0.351012072	1062.130378	1354.69957	Mus musculus v-maf musculoaponeurotic fibrosarcoma oncogene family, protein B (avian) (Mafb), mRNA [NM_010658]
Slit diaphragm	*Nphs1*	0.241760054	30.36086194	35.89972527	Mus musculus nephrosis 1, nephrin (Nphs1), mRNA [NM_019459]
	Nphs2	-0.960866934	47.81448206	24.56459945	Mus musculus nephrosis 2, podocin (Nphs2), mRNA [NM_130456]
	Cd2ap	-0.115950295	17700.56609	16333.62721	Mus musculus CD2-associated protein (Cd2ap), mRNA [NM_009847]
	Plce1	0.248835325	1088.072842	1292.899798	Mus musculus phospholipase C, epsilon 1 (Plce1), mRNA [NM_019588]
	Cadh3	0.011372427	5210.825285	5252.063322	Mus musculus cadherin 3 (Cdh3), transcript variant 1, mRNA [NM_001037809]
Actin cytoskelton	*Rhoa*	0.090664734	6615.354267	7044.430862	Mus musculus ras homolog gene family, member A (Rhoa), mRNA [NM_016802]
	Rac1	-0.037604771	35745.07937	34825.39905	Mus musculus RAS-related C3 botulinum substrate 1 (Rac1), mRNA [NM_009007]
	Cdc42	-0.019192871	54004.91873	53291.22297	Mus musculus cell division cycle 42 (Cdc42), transcript variant 1, mRNA [NM_009861]
	Actn4	0.120985915	48879.47325	53155.34077	Mus musculus actinin alpha 4 (Actn4), mRNA [NM_021895]
	Myo1e	0.024558677	5286.710284	5377.475122	Mus musculus myosin IE (Myo1e), mRNA [NM_181072]
	Myh9	0.242171158	117688.0098	139198.0019	Mus musculus myosin, heavy polypeptide 9, non-muscle (Myh9), mRNA [NM_022410]
	Inf2	-0.074537313	47479.49345	45088.74113	Mus musculus inverted formin, FH2 and WH2 domain containing (Inf2), mRNA [NM_198411]
	Synpo	0.175435065	15456.2313	17454.79791	Mus musculus synaptopodin (Synpo), transcript variant A, mRNA [NM_177340]
	Dnm1	0.396818966	13597.78851	17902.87148	Mus musculus dynamin 1 (Dnm1), mRNA [NM_010065]
	Ctsl	0.268807025	91064.41573	109715.4233	Mus musculus cathepsin L (Ctsl), mRNA [NM_009984]
	Itga3	0.035540042	8217.144522	8422.083521	Mus musculus integrin alpha 3 (Itga3), mRNA [NM_013565]
	Itgb1	0.333834759	2221.072816	2799.349173	Mus musculus integrin beta 1 (fibronectin receptor beta) (Itgb1), mRNA [NM_010578]
	Ilk	0.001490658	24832.1821	24857.8531	Mus musculus integrin linked kinase (Ilk), transcript variant 1, mRNA [NM_001161724]
	Nck1	-0.048948456	4074.054995	3938.147095	Mus musculus non-catalytic region of tyrosine kinase adaptor protein 1 (Nck1), mRNA [NM_010878]
Cell surface molecule	*Podxl*	-0.281238708	608.4762259	500.70546	Mus musculus podocalyxin-like (Podxl), mRNA [NM_013723]
Cell signal	*Mtor*	-0.058779787	1161.030662	1114.677437	Mus musculus mechanistic target of rapamycin (serine/threonine kinase) (Mtor), mRNA [NM_020009]
	Ctsl	0.268807025	91064.41573	109715.4233	Mus musculus cathepsin L (Ctsl), mRNA [NM_009984]
	Gsk3b	-0.044723504	1552.440294	1505.052988	Mus musculus glycogen synthase kinase 3 beta (Gsk3b), mRNA [NM_019827]
	Akt1	0.043890798	37880.14105	39050.26939	Mus musculus thymoma viral proto-oncogene 1 (Akt1), transcript variant 1, mRNA [NM_009652]
	Nfkb1	-0.192913706	1579.866923	1382.126183	Mus musculus nuclear factor of kappa light polypeptide gene enhancer in B cells 1, p105 (Nfkb1), mRNA [NM_008689]
	Tgfb1	0.139616781	763.9513433	841.5783177	Mus musculus transforming growth factor, beta 1 (Tgfb1), mRNA [NM_011577]
	Vegfa	-0.053569238	678.5666319	653.8325723	Mus musculus vascular endothelial growth factor A (Vegfa), transcript variant 3, mRNA [NM_001025257]
	Notch1	-0.127652906	69.45945238	63.57758118	Mus musculus notch 1 (Notch1), mRNA [NM_008714]
	Notch2	0.07352247	6899.884867	7260.630121	Mus musculus notch 2 (Notch2), mRNA [NM_010928]
Apoptosis	*Fas*	-0.057497238	9741.358286	9360.760281	Mus musculus Fas (TNF receptor superfamily member 6) (Fas), transcript variant 1, mRNA [NM_007987]
	Bcl2	0.133346274	2013.408559	2208.37661	Mus musculus B cell leukemia/lymphoma 2 (Bcl2), transcript variant 2, mRNA [NM_177410]
	Bcl2l1	-0.060139588	118592.9281	113750.9342	Mus musculus BCL2-like 1 (Bcl2l1), transcript variant 1, mRNA [NM_001289716]
	Bax	0.012611815	41334.40988	41697.33284	Mus musculus BCL2-associated X protein (Bax), mRNA [NM_007527]
	Bak1	0.039906895	15473.05859	15907.03888	Mus musculus BCL2-antagonist/killer 1 (Bak1), mRNA [NM_007523]
	Casp9	-0.051652332	7779.944108	7506.328657	Mus musculus caspase 9 (Casp9), transcript variant 1, mRNA [NM_015733]
	Casp3	0.096449792	704.1817373	752.8683466	Mus musculus caspase 3 (Casp3), transcript variant 2, mRNA [NM_009810]
	Chek1	0.260269253	44.42363315	53.20628375	Mus musculus checkpoint kinase 1 (Chek1), mRNA [NM_007691]
Cell cycle	*Cdkn1c*	-0.052949856	1947.353274	1877.17711	Mus musculus cyclin-dependent kinase inhibitor 1C (P57) (Cdkn1c), transcript variant 2, mRNA [NM_009876]
	Cdkn1a	-0.020040506	45829.92524	45197.7021	Mus musculus cyclin-dependent kinase inhibitor 1A (P21) (Cdkn1a), transcript variant 1, mRNA [NM_007669]
	Cdkn1b	0.035535188	13993.11771	14342.06363	Mus musculus cyclin-dependent kinase inhibitor 1B (Cdkn1b), mRNA [NM_009875]
	Trp53	0.150703823	665.1088191	738.3445568	Mus musculus transformation related protein 53 (Trp53), transcript variant 1, mRNA [NM_011640]
	Cdk1	-0.28325155	40.6870756	33.4340712	Mus musculus cyclin-dependent kinase 1 (Cdk1), mRNA [NM_007659]
	Cdk2	-0.044956939	1033.698912	1001.983735	Mus musculus cyclin-dependent kinase 2 (Cdk2), transcript variant 1, mRNA [NM_183417]
	Cdk5	-0.000444298	8471.297831	8468.689381	Mus musculus cyclin-dependent kinase 5 (Cdk5), mRNA [NM_007668]
	Ccnd1	-0.033528252	7552.760056	7379.257717	Mus musculus cyclin D1 (Ccnd1), mRNA [NM_007631]
	Ccna2	0.059260139	2906.941894	3028.83371	Mus musculus cyclin A2 (Ccna2), mRNA [NM_009828]
	Ccnb1	-0.013942677	209.4022375	207.3882534	Mus musculus cyclin B1 (Ccnb1), mRNA [NM_172301]
	Ccne1	-0.055401656	299.8488117	288.5524537	Mus musculus cyclin E1 (Ccne1), mRNA [NM_007633]

Suppl Table 1.
