# Supplementary material for: Transcription factor 21 expression in injured podocytes of glomerular diseases
Source: Sci Rep. 2020 Jul 13;10:11516. doi: 10.1038/s41598-020-68422-3 (PMC7359327; doi:10.1038/s41598-020-68422-3)
Supplement: Supplementary file 2 — The specificity of polyclonal anti-TCF21 antibody. To verify the specificity of polyclonal anti-TCF21 antibody, we stained with the antibody supernatant reacted with recombinant human TCF21 peptide antigen. a: Reaction with anti-TCF21 antibody in the MGN sample. b: Negative control, immunohistochemistry without primary antibody reaction on the serial section. c: Antibody-absorption test using recombinant TCF21 peptide antigen. In the section using supernatant absorbed by TCF21 antigen, the stain was not found, comparing to positive stain with no-absorbing primary antibody (a). (PDF 889 kb) [file 41598_2020_68422_MOESM2_ESM.pdf]

Transcription factor 21 expression in injured podocytes of glomerular diseases

Joichi Usui[1,3], Misa Yaguchi[1], Satoshi Yamazaki[2,4], Mayumi Takahashi-Kobayashi[1], Tetsuya Kawamura[1], Shuzo Kaneko[1], Surya V Seshan[3], Pierre Ronco[5], Kunihiro Yamagata[1]

[1]Department of Nephrology and [2]Division of Stem Cell Therapy, Faculty of Medicine, University of Tsukuba, Tsukuba. Ibaraki, 305-8576, Japan.

[3]Department of Pathology and Laboratory Medicine, Weill Cornell Medicine, NY, NY, 10065, USA.

[4]Devision of Stem Cell Therapy, Distinguished Professor Units, The Institute of Medical Science, The University Tokyo, Tokyo, 108-8639, Japan.

[5]SorbonneUniversité, Université Pierre et Marie Curie Paris 06, and Institut National de la Santé et de la Recherche Médicale, Unité Mixte de Recherche S1155, Paris, France.

*Corresponding author: Joichi Usui, M.D., Ph.D.

Department of Nephrology, Faculty of Medicine, University of Tsukuba

1-1-1 Tennodai, Tsukuba, Ibaraki 3058575, Japan

TEL&FAX: +81-29-853-3202

E-MAIL: j-usui@md.tsukuba.ac.jp

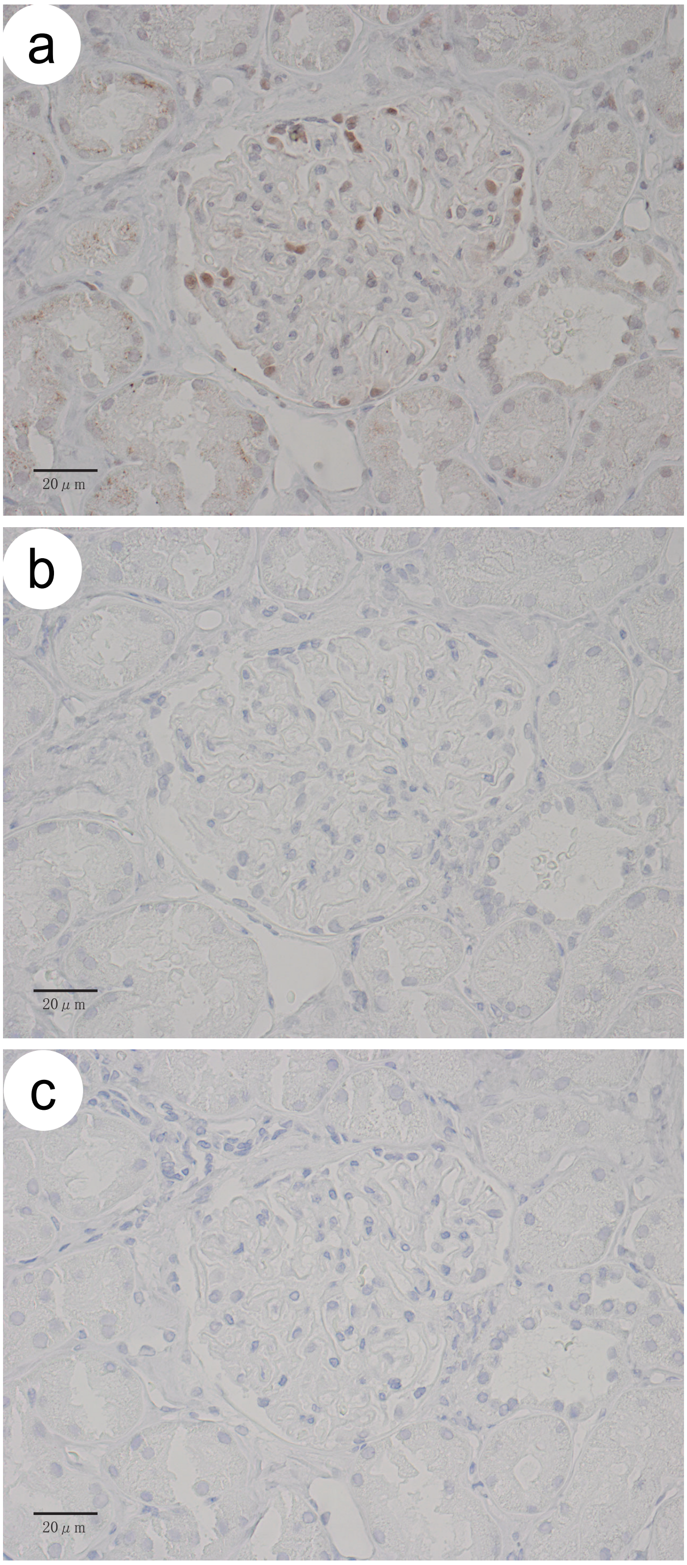

Usui J et al. Suppl Figure 1
