# Supplementary material for: Transcription factor 21 expression in injured podocytes of glomerular diseases
Source: Sci Rep. 2020 Jul 13;10:11516. doi: 10.1038/s41598-020-68422-3 (PMC7359327; doi:10.1038/s41598-020-68422-3)
Supplement: Supplementary file 3 — Histological analysis of TCF21 in human kidney biopsy samples with low power-magnification view. a: In NGD sample, TCF21 was mainly and weakly expressed in podocytes of glomeruli. By contrast, TCF21 expression was rarely seen in the tubulointerstitial area. The positive reaction of tubular cells was unremarkable. b: In MGN sample, TCF21 was also mainly expressed in podocytes of glomeruli, comparing to tubulointerstitial cells. (PDF 429 kb) [file 41598_2020_68422_MOESM3_ESM.pdf]

Transcription factor 21 expression in injured podocytes of glomerular diseases

Joichi Usui[1,3], Misa Yaguchi[1], Satoshi Yamazaki[2,4], Mayumi Takahashi-Kobayashi[1], Tetsuya Kawamura[1], Shuzo Kaneko[1], Surya V Seshan[3], Pierre Ronco[5], Kunihiro Yamagata[1]

[1]Department of Nephrology and [2]Division of Stem Cell Therapy, Faculty of Medicine, University of Tsukuba, Tsukuba. Ibaraki, 305-8576, Japan.

[3]Department of Pathology and Laboratory Medicine, Weill Cornell Medicine, NY, NY, 10065, USA.

[4]Devision of Stem Cell Therapy, Distinguished Professor Units, The Institute of Medical Science, The University Tokyo, Tokyo, 108-8639, Japan.

[5]SorbonneUniversité, Université Pierre et Marie Curie Paris 06, and Institut National de la Santé et de la Recherche Médicale, Unité Mixte de Recherche S1155, Paris, France.

*Corresponding author: Joichi Usui, M.D., Ph.D.

Department of Nephrology, Faculty of Medicine, University of Tsukuba

1-1-1 Tennodai, Tsukuba, Ibaraki 3058575, Japan

TEL&FAX: +81-29-853-3202

E-MAIL: j-usui@md.tsukuba.ac.jp

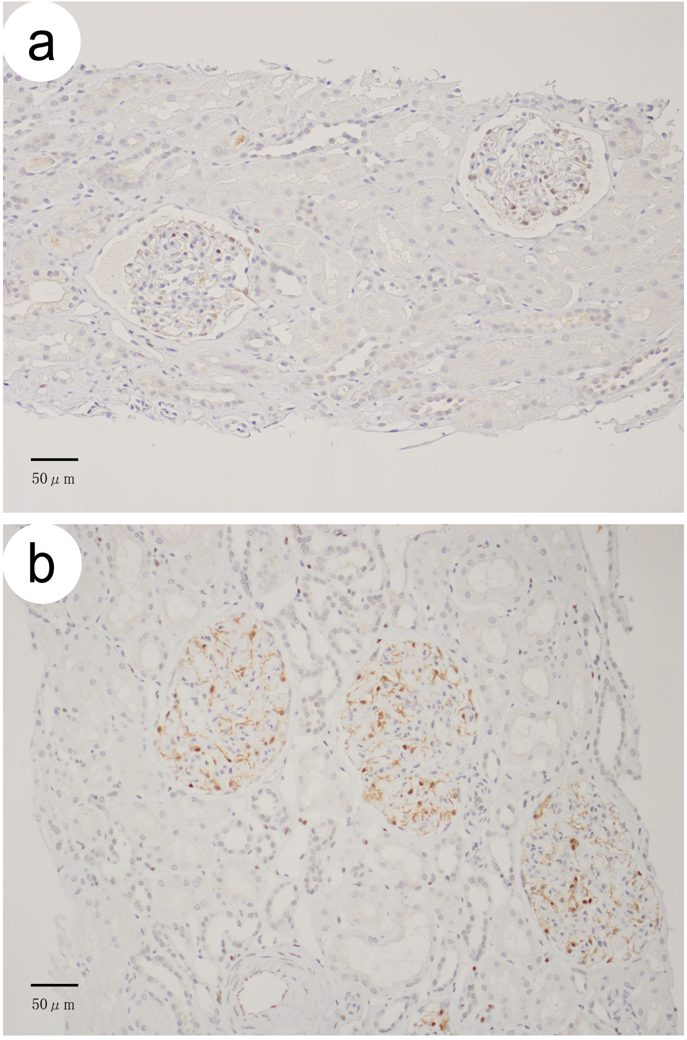

Usui J et al. Suppl Figure 2
