# Supplementary material for: Transcription factor 21 expression in injured podocytes of glomerular diseases
Source: Sci Rep. 2020 Jul 13;10:11516. doi: 10.1038/s41598-020-68422-3 (PMC7359327; doi:10.1038/s41598-020-68422-3)
Supplement: Supplementary file 4 — TCF21 expression data from Nephroseq datasets (Nephroseq.org). a: TCF21 expression in normal human renal tissues, comparison between glomeruli and tubulointerstitium (n=12, Fold Change:-62.414, p=8.48E-12; Lindenmeyer Normal Tissue Panel, Nephroseq.org). Sample size (n) is indicated in parenthesis. b: TCF21 expression in Healthy Living Donor (n=21), Membranous Glomerulonephropathy (n=21) and Minimal Change Disease (n=14) (modified from Ju CKD Glom, Nephroseq.org). (PDF 131 kb) [file 41598_2020_68422_MOESM4_ESM.pdf]

Transcription factor 21 expression in injured podocytes of glomerular diseases

Joichi Usui[1,3], Misa Yaguchi[1], Satoshi Yamazaki[2,4], Mayumi Takahashi-Kobayashi[1], Tetsuya Kawamura[1], Shuzo Kaneko[1], Surya V Seshan[3], Pierre Ronco[5], Kunihiro Yamagata[1]

[1]Department of Nephrology and [2]Division of Stem Cell Therapy, Faculty of Medicine, University of Tsukuba, Tsukuba. Ibaraki, 305-8576, Japan.

[3]Department of Pathology and Laboratory Medicine, Weill Cornell Medicine, NY, NY, 10065, USA.

[4]Devision of Stem Cell Therapy, Distinguished Professor Units, The Institute of Medical Science, The University Tokyo, Tokyo, 108-8639, Japan.

[5]SorbonneUniversité, Université Pierre et Marie Curie Paris 06, and Institut National de la Santé et de la Recherche Médicale, Unité Mixte de Recherche S1155, Paris, France.

*Corresponding author: Joichi Usui, M.D., Ph.D.

Department of Nephrology, Faculty of Medicine, University of Tsukuba

1-1-1 Tennodai, Tsukuba, Ibaraki 3058575, Japan

TEL&FAX: +81-29-853-3202

E-MAIL: j-usui@md.tsukuba.ac.jp

a

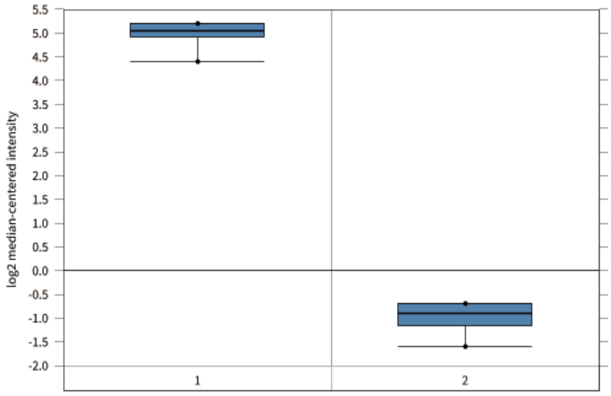

1. Glomeruli (6) 2. Tubulointerstitium (6)

b

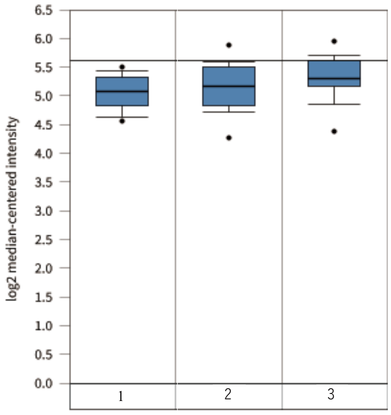

1. Healthy Living Donor (21)
2. Membranous Glomerulonephropathy (21)
3. Minimal Change Disease (14)

Usui J et al. Suppl Figure 3
