# Supplementary material for: Transcription factor 21 expression in injured podocytes of glomerular diseases
Source: Sci Rep. 2020 Jul 13;10:11516. doi: 10.1038/s41598-020-68422-3 (PMC7359327; doi:10.1038/s41598-020-68422-3)
Supplement: Supplementary file 7 — Comparison of Tcf21 (a), Rock1 (b) and Baiap2 (c) mRNA expression between Tcf21-MPs versus Control-MPs treated with ADR. a: Tcf21 constantly expressed in Tcf21-MPs with up-regulation trend (p=0.01). b: Rock1 expression of Control-MPs significantly decreased after ADR treatment (p=0.03). The difference of Rock1 between Tcf21-MPs and Control-MPs at pre-treatment permissive condition disappeared at 6 and 12 hours after ADR treatment (p=0.4, 0.4, respectively). C: Baiap2 expression of both MPs significantly decreased after ADR treatment (p<0.01, <0.01, respectively). The difference of Baiap2 between Tcf21-MPs and Control-MPs at pre-treatment permissive condition disappeared at 6 and 12 hours after ADR treatment (p>0.9, 0.2, respectively). Statistical analysis was performed with the Kruskal-Wallis test or Mann Whitney U test. (PDF 76 kb) [file 41598_2020_68422_MOESM7_ESM.pdf]

Transcription factor 21 expression in injured podocytes of glomerular diseases

Joichi Usui[1,3], Misa Yaguchi[1], Satoshi Yamazaki[2,4], Mayumi Takahashi-Kobayashi[1], Tetsuya Kawamura[1], Shuzo Kaneko[1], Surya V Seshan[3], Pierre Ronco[5], Kunihiro Yamagata[1]

[1]Department of Nephrology and [2]Division of Stem Cell Therapy, Faculty of Medicine, University of Tsukuba, Tsukuba. Ibaraki, 305-8576, Japan.

[3]Department of Pathology and Laboratory Medicine, Weill Cornell Medicine, NY, NY, 10065, USA.

[4]Devision of Stem Cell Therapy, Distinguished Professor Units, The Institute of Medical Science, The University Tokyo, Tokyo, 108-8639, Japan.

[5]SorbonneUniversité, Université Pierre et Marie Curie Paris 06, and Institut National de la Santé et de la Recherche Médicale, Unité Mixte de Recherche S1155, Paris, France.

*Corresponding author: Joichi Usui, M.D., Ph.D.

Department of Nephrology, Faculty of Medicine, University of Tsukuba

1-1-1 Tennodai, Tsukuba, Ibaraki 3058575, Japan

TEL&FAX: +81-29-853-3202

E-MAIL: j-usui@md.tsukuba.ac.jp

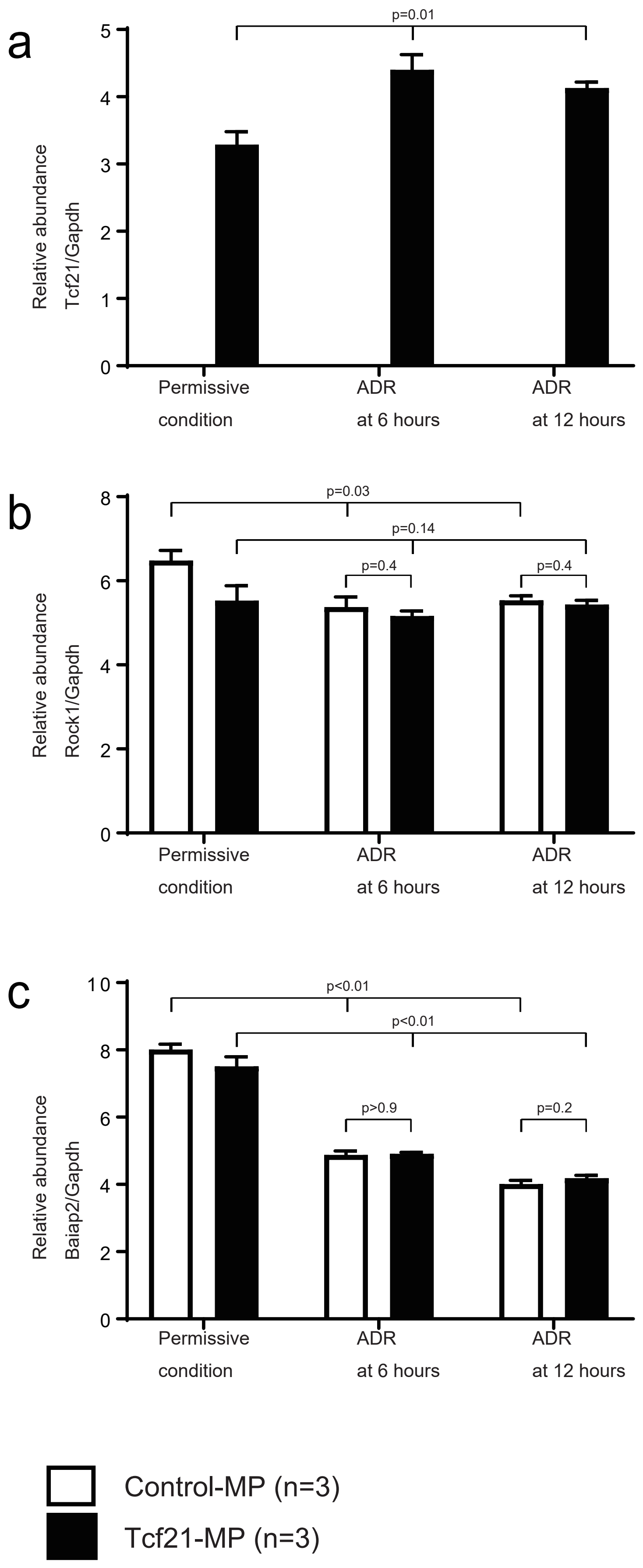

Usui J et al. Suppl Figure 6
